# Draft Whole-Genome Sequences of 25 *Salmonella enterica* Strains Representing 24 Serovars

**DOI:** 10.1128/genomeA.01718-15

**Published:** 2016-03-03

**Authors:** Catherine Yoshida, Stephanie L. Brumwell, Erika J. Lingohr, Aaminah Ahmad, Travis M. Blimkie, Benjamin A. Kogan, Jessica Pilsworth, Muhammad A. Rehman, Krista L. Schleicher, Jenitta Shanmugaraj, Andrew M. Kropinski, John H. E. Nash

**Affiliations:** a National Microbiology Laboratory at Guelph, Infectious Disease Prevention and Control Branch, Public Health Agency of Canada, Government of Canada, Guelph, Ontario, Canada; b Department of Pathobiology, Ontario Veterinary College, University of Guelph, Guelph, Ontario, Canada

## Abstract

We report the draft genome sequences of 25 *Salmonella enterica* strains representing 24 different serotypes, many of which were not available in public repositories during our selection process. These draft genomes will provide useful reference for the genetic variation between serotypes and aid in the development of molecular typing tools.

## GENOME ANNOUNCEMENT

*Salmonella* spp. are Gram-negative bacteria commonly responsible for human diarrheal illness through the contamination of foods including meats and produce. There are an estimated 1.7 million incidents of salmonellosis each year in North America alone, with over 80 million occurring globally (1). Having the ability to attribute illness to specific serotypes and subtypes can aid outbreak detection and source tracking. There is a momentum toward the acceptance and integration of new molecular methods for pathogen detection and classification. This places great importance on the availability of unique whole-genome sequences to accurately differentiate serotypes and subtypes and to validate methodologies. Genome sequences facilitate advancements in the detection and characterization of *Salmonella* serotypes, and those presented here contributed to the development of the *Salmonella* genoserotyping array (SGSA), a molecular-based serotyping assay (2), as well as a web-based platform for *in silico* serotyping of draft genome assemblies of *Salmonella* spp. (https://lfz.corefacility.ca/sistr-app; 3). The SGSA is able to provide the antigenic formula and serovar, preserving the nomenclature of legacy data gathered using traditional serotyping methods.

When this study began, there were numerous *Salmonella* serogroups and serovars not represented in GenBank, and isolates were selected to create a more complete public data set and expand the identification capacity of the SGSA. While this manuscript was being readied for publication, several of these serogroups and/or serovars became publicly available; however these will contribute to the accumulation of multiple representative genomes for the development of molecular typing tools. As of 10 July 2015, this is the first assembly and publication of whole-genome sequences for three *Salmonella* serogroups, O:43(U), O:56, and O:63, and 10 serovars: Blegdam, Fresno, Hillingdon, Itami, Milwaukee, Moscow, Virginia, Weslaco, subsp. *salamae* (II) 56:z_10_:e,n,x, and subsp. *arizonae* (IIIa) 63:g,z_51_:-.

The genomic DNA required for sequencing was extracted from *Salmonella* isolates grown overnight at 37°C on Luria-Bertani agar (BD Canada, Mississauga, ON, Canada) using an EZ1 DNA tissue kit (Qiagen Ltd., Mississauga, ON, Canada). Three technologies were used for sequencing: Roche’s 454 GS-FLX Titanium, obtaining an average coverage of 40-fold; Illumina HiSeq 2000 with TruSeq sample preparation of 2 × 100 paired-end runs, obtaining an average coverage of 90-fold; and Illumina MiSeq with TruSeq sample preparation of 2 × 251 paired-end runs, obtaining an average coverage of 90-fold. Of the 25 draft genomes, 20 isolates were sequenced using both Illumina and 454 technologies, while five were sequenced by 454 only. Mira assembler version 4.0 (4) was used to assemble the reads into contigs, which were then proofread and corrected in the program Gap5 of the Staden software package (5). The genomes were annotated using the NCBI Prokaryotic Genome Annotation Pipeline (http://www.ncbi.nlm.nih.gov/genome/annotation_prok).

These draft genomes can provide useful information for the development and validation of molecular diagnostic tools, as well as other research activities.

### Nucleotide sequence accession numbers.

The draft genome sequences for these 25 *Salmonella* isolates have been deposited in DDBJ/ENA/GenBank under Bioproject no. PRJNA294295. The GenBank accession numbers are listed in Table 1. The raw sequence data are available in the Sequence Read Archive (SRA).

**TABLE 1:** *Salmonella* strains sequenced in this study

Serovar	Antigenic formula	Isolate no.	Sequencing method(s)^a^	Accession no.
Blegdam	9,12:g,m,q: -	SA20065575	1	LHSP00000000
Enteritidis	1,9,12:g,m,p: -	SA20093032	1, 2	LHSQ00000000
Enteritidis	1,9,12:f,g,m,t: -	SA20103550	1, 2	LHSR00000000
Fresno	9,46:z_38_: -	ST224	1, 2	LHSS00000000
Gallinarum	1,9,12: -: -	ST572	1, 2	LHST00000000
Gaminara	16:d: 1,7	SA20063285	1, 2	LHSU00000000
Hadar	6,8:z_10_:e,n,x	SA20026260	1, 2	LHSV00000000
Hillingdon	9,46:g,m: -	S01-0588	1	LHSX00000000
Hvittingfoss	16:b:e,n,x	SA20014981	1, 2	LHSW00000000
Itami	9,12: l,z_13_: 1,5	SA20014991	1, 3	LHSY00000000
Johannesburg	40:b:e,n,x	SA20025782	1	LHSZ00000000
Kentucky	8,20:i:z_6_	SA20030505	1, 2	LHTA00000000
Manhattan	6,8:d: 1,5	SA20034532	1, 2	LHTB00000000
Milwaukee	43:f,g,[t]: -	SA19950795	1, 2	LHTC00000000
Moscow	9,12:g,q: -	SA20061414	1	LIXO00000000
Newport	6,8,20:e,h: 1,2	L0167	1	LIXP00000000
Panama	1,9,12:l,v: 1,5	SA20030878	1, 3	LHTD00000000
Paratyphi_A	2,12:a: -	SA19950809	1, 2	LHTE00000000
Pullorum	1,9,12: -: -	S4037-07	1, 2	LHTF00000000
Rubislaw	11:r:e,n,x	SA20030553	1, 2	LHTG00000000
subsp. II 56:z10:e,n,x	56:z_10_:e,n,x	1369–1373	1, 2	LHTH00000000
subsp. IIIa 62:z36: -	62:z_36_: -	5335/86	1, 2	LHTK00000000
subsp. IIIa 63:g,z51: -	63:g,z_51_: -	So 20/20	1, 2	LHTL00000000
Virginia	8:d: 1,2	SA19971529	1, 2	LHTI00000000
Weslaco	42:z_36_: -	247K	1, 3	LHTJ00000000

^a^ 1 = Roche 454 GS-FLX titanium; 2 = Illumina MiSeq; 3 = Illumina HiSeq.

## References

[1] Majowicz SE, Musto J, Scallan E, Angulo FJ, Kirk M, O’Brien SJ, Jones TF, Fazil A, Hoekstra RM 2010 The global burden of nontyphoidal *Salmonella* gastroenteritis. Food Safety 50:882–889. doi:10.1086/650733. 20158401

[2] Franklin K, Lingohr EJ, Yoshida C, Anjum M, Bodrossy L, Clark CG, Kropinski AM, Karmali MA 2011 Rapid genoserotyping tool for classification of *Salmonella* serovars. J Clin Microbiol 49:2954–2965. doi:10.1128/JCM.02347-10. 21697324 PMC3147765

[3] Yoshida CE, Kruczkiewicz P, Laing CR, Lingohr EJ, Gannon VP, Nash JH, Taboada EN 2016 The *Salmonella* *In Silico* Typing Resource (SISTR): An Open Web-Accessible Tool for Rapidly Typing and Subtyping Draft *Salmonella* Genome Assemblies. PLoS One 11(1):e0147101. doi:10.1371/journal.pone.0147101. 26800248 PMC4723315

[4] Chevreux B, Wetter T, Suhai S 1999 Genome sequence assembly using trace signals and additional sequence information, p 45–56. *In* Computer science and biology. Proceedings of the German Conference on Bioinformatics, GCB '99. GCB, Hannover, Germany.

[5] Bonfield JK, Whitwham A 2010 Gap5—editing the billion fragment sequence assembly. Bioinformatics 26:1699–1703. doi:10.1093/bioinformatics/btq268. 20513662 PMC2894512

